# Evaluating the Solvent Stark Effect from Temperature‐Dependent Solvatochromic Shifts of Anthracene

**DOI:** 10.1002/cphc.202000010

**Published:** 2020-02-28

**Authors:** Timais Janz, Manuel Güterbock, Fabian Müller, Martin Quick, Ilya N. Ioffe, Florian A. Bischoff, Sergey A. Kovalenko

**Affiliations:** ^1^ Department of Chemistry Humboldt-Universität zu Berlin Brook-Taylor-Str. 2 D-12489 Berlin Germany; ^2^ Department of Chemistry Lomonosov Moscow State University Moscow Russia

**Keywords:** anthracene, computational chemistry, solvatochromic shifts, solvent Stark effect, solute polarizabilities

## Abstract

The solvent Stark effect on the spectral shifts of anthracene is studied with temperature‐dependent solvatochromic measurements. The Stark contribution Δ*v*_Stark_ to the absorption shift Δ*v*_p_ in polar solvents is measured to be Δ*v*_Stark_=(53±35) cm^−1^, in reasonable agreement with dielectric continuum theory estimate of 28 cm^−1^, whereas the major shift Δ*v*_p_∼300 cm^−1^ presumably originates from the solute quadrupole. We pay attention to the accurate correction of Δ*v*_p_ for the nonpolar contribution that is crucial when the shifts are modest in magnitude.

## Graphical abstract

**The solvatochromic Stark shift** from anthracene in polar solvents is determined, Δ*v*_Stark_=(53±35) cm^−1^. It agrees with dielectric continuum theory estimate of 28 cm^−1^. The temperature dependence allows one to isolate Δ*v*_Stark_ from a much larger shift of ∼300 cm^−1^ usually observed for nondipolar solutes in polar solvents, which is presumably due to the solute quadrupole.

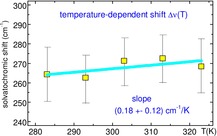

## 1 Introduction

When a nondipolar polarizable solute like anthracene, perylene or trans‐stilbene is immersed in a polar solvent, it is stabilized via interaction with fluctuating solvent electric field ϵ by energy $E_{\alpha }=-\alpha \left\langle {\;\epsilon }^{2}\;\right\rangle /2$ where α is the solute polarizability, and the averaging is taken over all solvent configurations.^1^, ^2^ This stabilization is commonly called the solvent Stark effect.^3^, ^4^, ^5^, ^6^, ^7^

Karlström and Halle^1^ applied a fluctuation approach for dielectrics with a spherical cavity of radius *a* to obtain the following exact result

(1) $$E_{\alpha }=-\frac{3}{2}RT\cdot \ln\left[\left({1-\alpha_{l}\chi }_{l}^{q}\right)/\left(1-\alpha_{l}\chi_{l}\right)\;\right]$$

(2) $$\chi_{l}=\frac{l!\left(l+1\right)\left(\epsilon -1\right)}{\left(l+1\right)\epsilon +l}\frac{1}{a^{3}}\;\chi_{l}^{q}=\frac{l!\left(l+1\right)\left(n^{2}-1\right)}{\left(l+1\right)n^{2}+l}\frac{1}{a^{3}}$$

Here multipolar susceptibilities $\chi_{l}$_,_ $\chi_{l}^{q}$ are related to slow (orientational) and fast (quantum, electronic) degrees of freedom of the solvent with dielectric constant ϵ and refractive index *n*; *R*=8.31 J/mol/K is the gas constant and *T* temperature; *l*=1 or 2 corresponds to dipolar or quadrupolar solute, or with *l* ≥3 to higher multipoles. For a nondipolar solute and when ${\alpha_{l}\chi }_{l}^{q}$ , $\alpha_{l}\chi_{l}\;$ ≪1, one gets(3)

(3) $$E_{\alpha }=-\frac{3}{2}RT\alpha_{1}\left(\chi_{1}-\chi_{1}^{q}\right)\;]=-\frac{3\alpha }{2a^{3}}RTf_{p}$$

where $f_{p}=[2(\epsilon -1)/(2\epsilon +1)\;-2(n^{2}-1)/(2n^{2}+1)]$ is the well‐known response in polar solvents.^4^ A very similar expression for $E_{\alpha }$ was also derived by Scaife.^2^

The Stark effect directly results in solvatochromic shifts for absorption(4)

(4) $${\Delta v}_{Stark}=-{\frac{3}{2}f}_{p}\frac{RT\left(\alpha_{e}^{FC}-\alpha_{g}\right)}{a^{3}}=-B_{Stark}f_{p}$$

where the polarizability difference appears because α differs in ground (g) and excited (e) electronic state of the solute; the superscript FC abbreviates “Franck‐Condon” indicating that α_e_ is taken at ground state (*S*_0_) chromophore geometry.

Hereinafter, we apply the following units: Δ*v* in cm^−1^, *a* in Å, α in Å^3^, dipole moment *μ* in D, quadrupole moment Q in DÅ. Energies *E* and shifts Δ*v* are in eV, kJ/mol or cm^−1^ with the relations between the units(5)

(5) $$1\;\mathrm{eV}=96.49\;\mathrm{kJ}/\mathrm{mol}=8065\;\mathrm{cm}{}^{-1}=1.602\;D{}^{2}/Å{}^{3},1\;D{}^{2}/Å{}^{3}=0.624\;\mathrm{eV}=5032\;\mathrm{cm}{}^{-1}\;$$

Just to give an idea of the expected shift (4), one has 3*f*_p_/2≈1, ${(\alpha }_{e}^{FC}-\alpha_{g})$ ∼15 Å^3^, *a*=5 Å, *RT=*2.44 kJ/mol=204 cm^−1^ at *T*=20 °C, that predicts quite a small value Δ*v*_Stark_ ∼20 cm^−1^.

In the late 1960s Baur and Nicol^8^ suggested a *different* expression for the Stark shift, ${\Delta v}_{Stark}∼\;\epsilon (\epsilon -1)/(2\epsilon +1)$ which gives for ϵ>10 a much larger shift than that by Eq. (4). Furthermore, they tried to ascribe the *full* observed shift from nondipolar solutes in polar solvents entirely to the Stark contribution, and even obtained a support from other workers.^9^ However, Ghoneim and Suppan^3^ experimentally demonstrated an inconsistency in their approach, and instead proposed quadrupolar or higher multipolar nature of the aforementioned shifts.

Since then, to the best of our knowledge, there were no attempts to quantify the Stark contribution to the solvatochromic shifts experimentally.

The aim of the present paper is to determine the weak Stark contribution to temperature‐dependent solvatochromic shifts of the absorption spectra of anthracene.

In addition, we propose a simple method for correcting the shifts for the nonpolar contribution, the correction being crucial when the shifts are modest in magnitude.

The paper is organized as follows. In section 2.1 we overview the theory of solvatochromic shifts and introduce the correction procedure, section 2.2 describes our calculations, followed by section 2.3 for the experimental results and discussion.

## 2 Results and Discussion

### 2.1 Solvatochromic Shifts

For a dipolar solute a classical theory^4^, ^10^, ^11^, ^12^, ^13^, ^14^ expresses absorption shifts Δ*v* as the sum of nonpolar Δ*v*_n_ and polar Δ*v*_p_ contribution(6), (7)

(6) $$\Delta v=\Delta v_{n}+\Delta v_{p}=-\frac{f_{n}}{2a^{3}}\left[\left({\left(\mu_{e}^{FC}\right)}^{2}-\mu_{g}^{2}\right)+C\left(\alpha_{e}^{FC}-\alpha_{g}\right)\right]-\frac{f_{p}}{a^{3}}\mu_{g}\left(\mu_{e}^{FC}-\mu_{g}\right)$$

(7) $$f_{n}=2(n^{2}-1)/(2n^{2}+1),\;f_{p}=[2(\epsilon -1)/(2\epsilon +1)\;-2(n^{2}-1)/(2n^{2}+1)]$$

Here *f*_n_, *f*_p_ is already familiar nonpolar and polar solvent response, *μ*, *α* is the solute dipole moment and polarizability, and the products of the dipole moments are to be understood as scalar products. The nonpolar part Δ*v*_n_ is proportional to *f*_n_ and consists of inductive (the first term) and dispersive contribution.^4^ The semiempirical constant *C* is often expressed via solute (*I*) and solvent (*I′*) ionization potential, $C=2II{}^{'}/\left(I+I{}^{'}\right)$ and usually is in the range of 10 eV=80000 cm^−1^. This gives an estimate of 4000 cm^−1^ for the dispersive term, and with *μ*_g_=5 D, *μ*_e_=10 D an estimate of 1500 cm^−1^ for the inductive term. Regarding the polar part Δ*v*_p_, it is proportional to *f*_p_ and represents the dipolar shift in polar solvents. With the above *μ*_g_=5 D, *μ*_e_=10 D, the dipolar shift Δ*v*_p_ reaches 1000 cm^−1^.

When like in our case, the solute dipoles vanish, *μ*_g_≈*μ*_e_≈0, the shifts Δ*v* are strongly dominated by the dispersive contribution, which exceeds the expected Stark shift by two orders of magnitude.

Baur and Nicol^8^ and Gerhold and Miller^9^ plotted experimental shifts Δ*v* against the calculated shifts given by a sum of Δ*v*_n_ and their Stark term ${\Delta v}_{Stark}∼\;\epsilon (\epsilon -1)/(2\epsilon +1)\cdot RT\left(\alpha_{e}^{FC}-\alpha_{g}\right)/a^{3}$ . It is however quite clear, that small (in percentages) errors in the huge dispersive contribution Δ*v*_n_ may completely mask the effect of interest.

We therefore apply below a different approach. First, we note that both Δ*v*_n_ and Δ*v*_p_ contribute to the shift Δ*v* in polar solvents. On the other hand in nonpolar solvents, Δ*v*_p_ vanishes completely since ϵ*=n* ^2^ and *f*_p_=0, and hence Δ*v*_n_ can be fully determined with a set of nonpolar solvents only(8)

(8) $$\Delta v_{n}=-\frac{f_{n}}{2a^{3}}C\left(\alpha_{e}^{FC}-\alpha_{g}\right)\equiv -B_{n}f_{n}$$

with the slope *B*_n_ being calculated from a linear fit of Δ*v*_n_ against *f*_n_. Having this result at hand one can get rid of the nonpolar contribution to Δ*v* (9)

(9) $$\Delta v_{p}=\Delta v-\Delta v_{n}=\Delta v+B_{n}\left(f_{n}-f_{pe}\right)$$

In our case Δ*v*_p_ does not contain the dipolar part, but presumably contains Stark Δ*v*_Stark_ and quadrupolar^3^ Δ*v*_Q_ part(10)

(10) $$\Delta v_{p}=-\frac{RT\left(\alpha_{e}^{FC}-\alpha_{g}\right)}{a^{3}}f_{p}-\frac{{2Q}_{gi}\left(Q_{ei}^{FC}-Q_{gi}\right)}{3a^{5}}f_{Q}\;\equiv -B_{Stark}f_{p}\;-\;B_{Q}f_{Q}$$

where the traceless quadrupole tensors Q are according to Buckingham^15^ and their products should be understood as the double inner products. The quadrupolar response *f*_Q_=[3(ϵ−1)/(3ϵ+2)−3(n^2^−1)/(3n^2^+2)] is slightly different from *f*_p_, but in the realm of more common solvents, where 1.4<*n* ^2^<2.6 (from perfluoroalkanes to CS_2_) and *n* ^2^≤ϵ<111 (formamide) *f*_Q_ deviates from *f*_p_ by at most 6 %, the highest discrepancy to be expected for extreme cases, perfluoroalkanes and compounds with high ϵ and *n* ^2^. Therefore, one can safely substitute *f*_p_ to obtain(11)

(11) $$\Delta v_{p}=-[\;\frac{{2Q}_{g}\left(Q_{e}^{FC}-Q_{g}\right)}{3a^{5}}+\frac{RT\left(\alpha_{e}^{FC}-\alpha_{g}\right)}{a^{3}}{\;]f}_{p}\;\equiv -[\;B_{Q}+B_{Stark}{\;]f}_{p}$$

In experiment the polar shifts Δ*v*_p_ from nondipolar chromophores are in *no* case negligible and reach 300 cm^−1^ for anthracene, stilbene or diphenylbutadiene. We believe, following Suppan,^3^ that these shifts originate from quadrupolar^15^ or higher multipolar contribution, as shall be discussed in detail in our forthcoming article.

Regarding the Stark contribution, although it is much smaller in magnitude, it can be derived from temperature‐dependent shifts Δ*v*_p_(T).

A further very helpful comparison is between Stark $B_{Stark}=RT\left(\alpha_{e}^{FC}-\alpha_{g}\right)/a^{3}$ and nonpolar *B*_n_=$C\left(\alpha_{e}^{FC}-\alpha_{g}\right)/2a^{3}$ slope. As seen, the both depend on the solute parameters in a similar fashion. This allows one to exclude the solute radius *a* (not well‐defined in the continuum dielectric theory) and to express *B*_Stark_ through the well‐measured quantity *B*_n_ that provides an improved estimate for the Stark shift. With $\left(\alpha_{e}^{FC}-\alpha_{g}\right)$ =16.5 Å^3^,^16^, ^17^, ^18^ and taking *I*=7.4 eV for anthracene, *I′*=10.4 eV for n‐pentane, one calculates *C*=69740 cm^−1^. And with our experimental *B*_n_=3150 cm^−1^ (see Figure 2) this gives at T=(12) K

(12) $$B{}_{\mathrm{Stark}}/B{}_{n}=3RT/C\;\approx 0.0088,\;B{}_{\mathrm{Stark}}=28\;\mathrm{cm}{}^{-1}$$

### 2.2 Calculations

Vacuum static polarizabilities for *S*_0_ and *S*_n_ states are computed with two approaches. The first uses the CC2 approximation of coupled‐cluster theory with a aug‐cc‐pVTZ basis set,^19^ and corresponding auxiliary basis set^20^ using the ricc2 module of the Turbomole program package version 7.0.2.^21^ The second approach involves the RI‐XMCQDPT2 quasi‐degenerate perturbation theory^22^ implemented in the Firefly V8.2 software^23^ which is partly based on the GAMESS(US) package.^24^ The perturbation corrections are applied on top of the CASSCF(14e,14o)/aug‐cc‐pwCVTZ reference where the active space encompasses all the 14 π‐orbitals. To suppress the intruder state effects, the intruder‐state‐avoidance (ISA) parameter is set 0.02 a.u.

The anthracene polarizabilities have already been calculated.^16^, ^17^, ^18^ Pavlovich^16^ considered the temperature‐dependent shifts of absorption in frozen glassy alcohols where the Stark effect and the dispersive contribution were added up. Mathies and Albrecht^17^ performed electric field perturbation spectroscopy in a frozen medium, and Bendkowsky et al.^18^ measured the quadratic Stark effect in jet‐cooled molecules.

Our XMCQDPT2 and RI‐CC2 computations confirm the first absorbing excited state to be indeed *S*_1_ dominated by the HOMO→LUMO excitation. At the CASSCF level it emerges incorrectly as *S*_3_. The second (after *S*_1_) bright absorbing state turns out to be *S*_6_ which is almost degenerate with *S*_5_ at the XMCQDPT2 level. It involves a mixture of several single excitations. Taking into account that the task of accurate description of the higher‐lying excited states would require at least further augmentation of the basis set, the XMCQDPT2 calculations were primarily focused at the task of more accurate description of the *S*_0_ and *S*_1_ states. In view of that, we use the CASSCF reference averaged over the five lowest singlet roots and include 13 states in the XMCQDPT2 model space. Resulting vertical gas‐phase excitation energies of 3.39 eV for *S*_0_→*S*_1_ and 4.88 eV for *S*_0_→S_6_ are in a good agreement with the experiment.

The calculated polarizabilities are given in Table 1 (x is the long axis of the anthracene molecule, y is the short one, and z – the perpendicular one). As seen Δα for *S*_0_→*S*_1_ equals 15.9 Å^3^, in agreement with both the present experiment (see below) and the earlier estimates.^16^, ^17^, ^18^ A very close result was previously obtained in the relaxed RI‐CC2/aug‐cc‐pVTZ calculations.^25^ At the same time, considering the *S*_0_ and *S*_1_ states separately, our values in Table 1 are lower than the previous CCSD(T) estimates for *S*_0_ 26 (interestingly, the discrepancy is almost entirely associated with α_xx_) and than the above RI‐CC2 data for *S*_1_.^25^) Unfortunately there is an understandable lack of reliable polarizability benchmarks even for the ground state of anthracene, as can be seen from a survey of the previous condensed‐phase experimental data.^18^

**Table 1:** Anthracene Polarizabilities [Å^3^].

	α_xx_	α_yy_	α_zz_	α
*S*_0_	34.2	24.1	12.8	23.7
*S*_1_	77.7	28.0	13.1	39.6
*S*_6_	90.7	22.8	12.8	42.1

α=(α_xx_+α_yy_+α_zz_)/3.

The XMCQDPT2 polarizability of the *S*_6_ state is considerably underestimated. This obviously results from the coupling to *S*_5_ which is placed by the calculation only a few meV below *S*_6_. Thus, an accurate computational treatment of *S*_6_ requires very precise energies of the other states and possibly even an explicit consideration of the relevant vibronic levels. Our present RI‐CC2 data are however qualitatively correct, suggesting a 40 % increase in the polarizability from *S*_1_ to *S*_6_.

### 2.3 Experimental Shifts and Discussion

**Figure 1:**
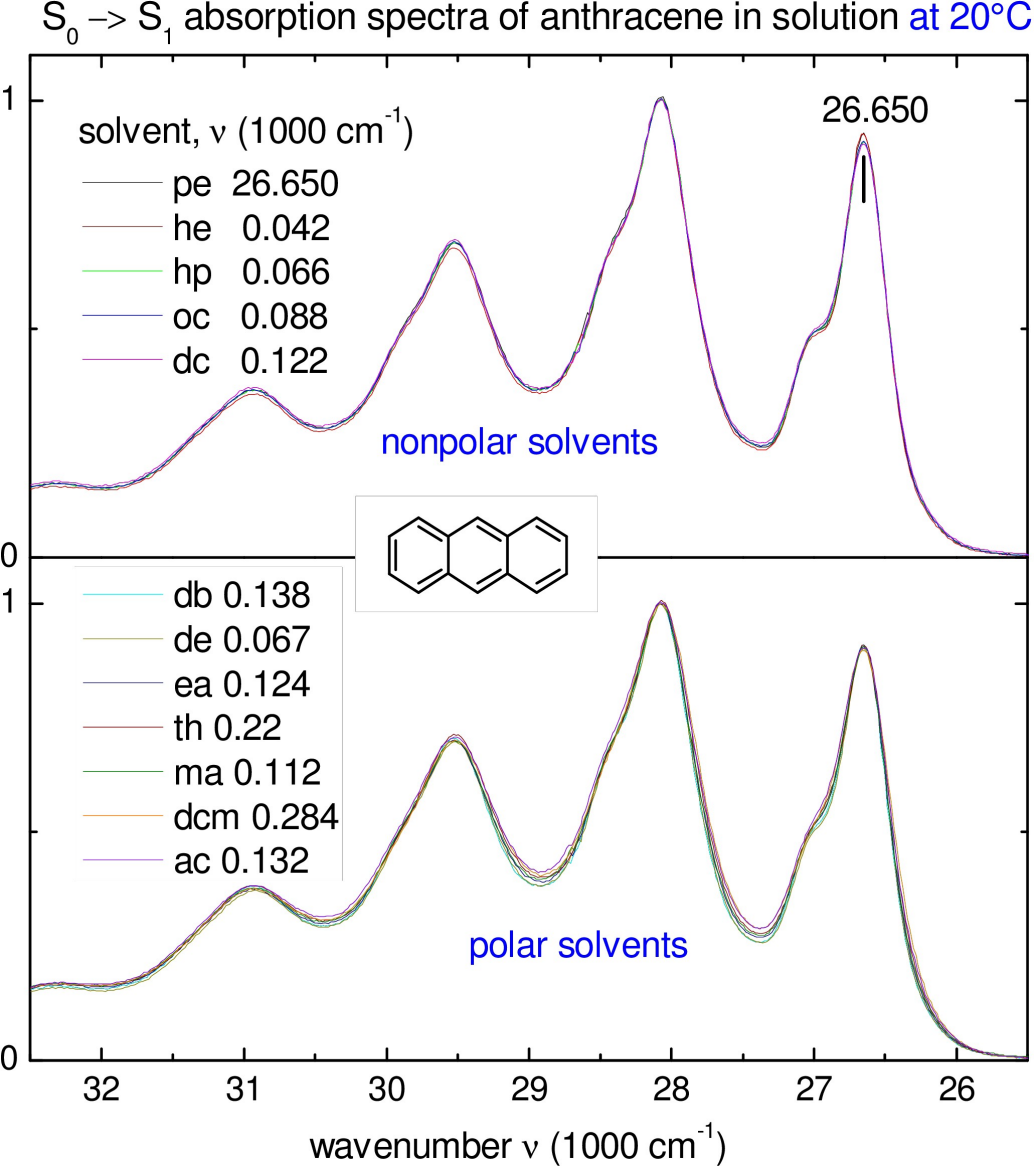
*S*_0_→*S*_1_ absorption spectra of anthracene at 20 °C in nonpolar and polar solvents, shifted for best coincidence with those in n‐pentane. The shifts are indicated in units of 1000 cm^−1^, the accuracy of the shifts is estimated ±1 cm^−1^. Full solvent names are given in Table 2.

Absorption spectra of anthracene in solution are recorded at *T*=10, 20, 30, 40, 50 °C with 0.02 nm step both in the visible (*S*_0_→*S*_1_) and in the UV (*S*_0_→*S*_6_). Anthracene is chosen as the probe because its narrow sub‐bands (see Figure 1) allow for high accuracy ±1 cm^−1^ of the spectral shifts in the visible. The nonpolar and polar solvents used are collected in Table 2 (see ref. 27 for the full solvent properties).

**Table 2:** Solvent Properties^27^ at T=25 °C.

	Solvent	ϵ	dϵ/dT/ϵ x1000	*n*	d*n*/dT x1000
1	2‐methyl‐butane (tm)	1.84	–	1.3537	–
2	n‐pentane (pe)	1.84	2.0	1.3547	0.552
3	n‐hexane (he)	1.88	1.9	1.3723	0.52
4	n‐heptane (hp)	1.92	1.68	1.3851	0.506
5	n‐octane (oc)	1.95	1.54	1.3951	0.476
6	n‐decane (dc)	1.99	1.5	1.4097	0.444
7	n‐dodecane (dd)	2.00	–	1.4195	–
8	n‐hexadecane (hd)	2.05	0.65	1.4325	–
9	cyclohexane (ch)	2.02	1.82	1.4235	0.538
10	di‐n‐butylether (db)	3.08	–	1.3968	0.45
11	di‐n‐propylether (dp)	3.39	–	1.381	
12	di‐n‐ethylether (de)	4.2	5.0	1.3495	0.56
13	ethylacetate (ea)	6.02	5.7	1.3698	0.49
14	tetrahydrofurane (th)	7.58	3.94	1.405	0.44
15	methylacetate (ma)	6.68	7.6	1.3589	0.50
16	acetonitrile (ac)	35.94	4.16	1.341	0.496
17	dichlorometane (dcm)	8.93	8.5	1.421	0.60
18	dimethysulfoxide (ds)	46.7	–	1.4783

Typical *S*_0_→*S*_1_ absorption spectra of anthracene are displayed in Figure 1. They consist of well resolved vibronic bands, with the 0–0 band peaked at 26650 cm^−1^ in n‐pentane. The spectra in nonpolar (top) and polar (bottom) solvents are shifted relative to n‐pentane for best coincidence in the red part, including the 0–0 and 0–1 band. We estimate the accuracy of such determined shifts to be ±1 cm^−1^. The shifts are indicated in units 1000 cm^−1^. Similarly, shifted UV spectra for the bright *S*_0_→*S*_6_ electronic transition are displayed in Figure S1, Supporting Information (SI).

**Figure 2:**
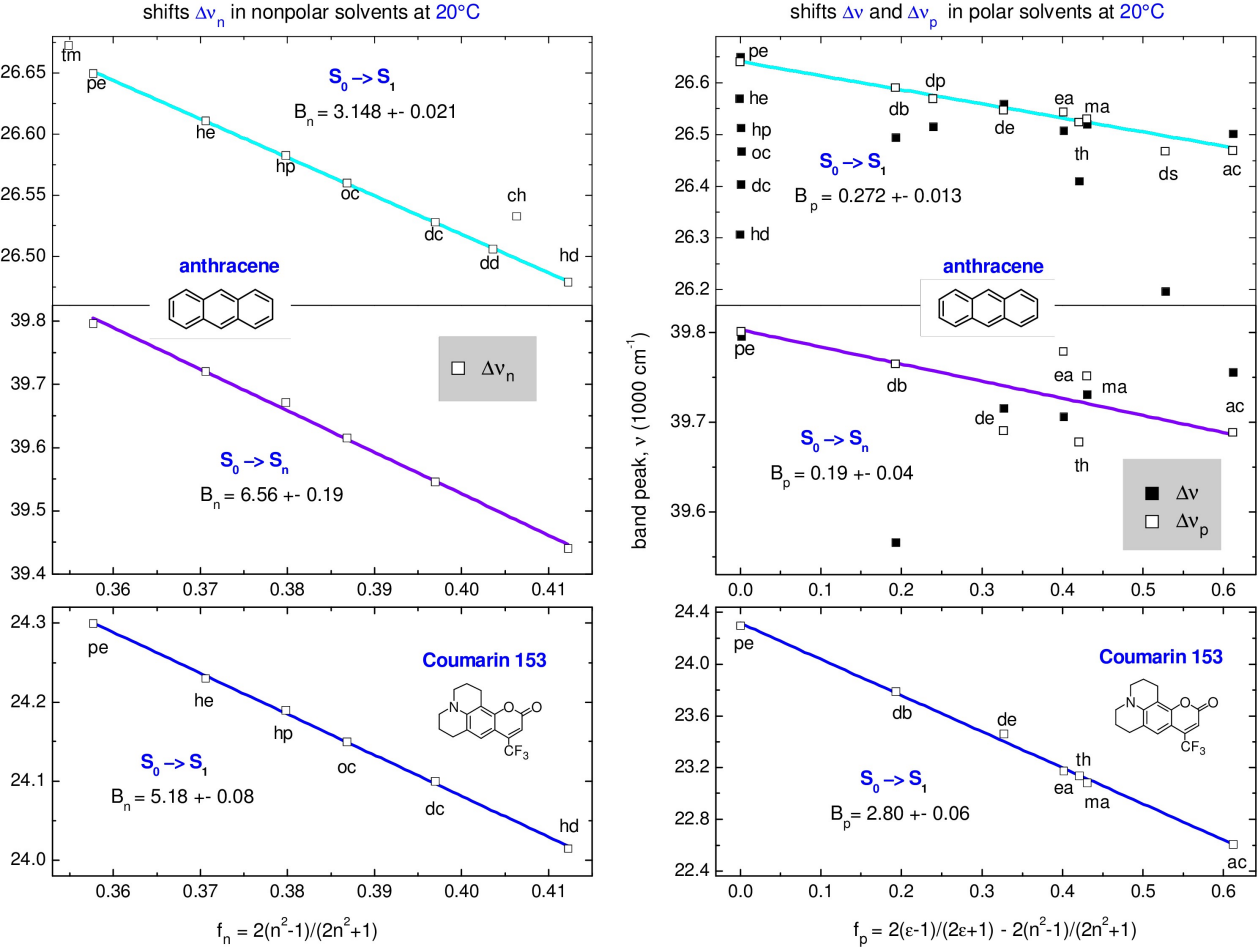
Solvatochromic shifts of anthracene in nonpolar solvents (Δ*v*_n_ at left), and in polar solvents (Δ*v*, Δ*v*_p_ at right) for *S*_0_→*S*_1_ and *S*_0_→*S*_n_ absorption (n=6 from our calculations). The shifts from highly polar C153 are shown for comparison at the bottom. Nonpolar and polar slopes *B*_n_, *B*_p_ from linear fits are given as inserts. For anthracene, a big scatter of directly measured shifts Δ*v* (black squares) in polar solvents is due to the nonpolar contribution Δ*v*_n_ (the point for tetrahydrofuran is out of the range). Its subtraction results in Δ*v*_p_ (open squares) which allow a much better fit than with original Δ*v* shown in Figure S3 (see Supporting Information). The solvents are listed in Table 1.

Figure 2 shows plots Δ*v*_n_(*f*_n_) and Δ*v*_p_(*f*_p_) for the *S*_0_→*S*_1_ and *S*_0_→*S*_6_ band of anthracene (top and middle frame), and for the *S*_0_→*S*_1_ band of highly polar dye C153.

Let us consider the *S*_0_→*S*_1_ band of anthracene first. Nonpolar shifts Δ*v*_n_ (top left) show a nice linear behavior along n‐hydrocarbons, from n‐pentane to n‐hexadecane. Note that 2‐methylbutane (tm) and cyclohexane (ch) apparently deviate from the linear fit. The deviation is systematic and is also observed with other solvatochromic probes.

Switching to polar solvents at right of Figure 2, one sees that directly measured shifts Δ*v* (black squares) reveal strong scatter that prevents from a satisfactory fit. As discussed above, this scatter is mainly due to the nonpolar contribution Δ*v*_n_=*B*_n_ *f*_n._ The subtraction (9) eliminates that contribution from Δ*v* and results in Δ*v*_p_ shown by the open squares. These allow now for a good linear fit with slope *B*_p_=(272±13)cm^−1^.

For comparison, the bottom frame of Figure 2 shows the shifts from highly polar C153.^28^ While its nonpolar slope *B*_n_=5180 cm^−1^ is comparable with that for anthracene, the polar slope *B*_p_=2800 cm^−1^ is by factor 10 larger, in which case the *f*_n_ contribution to Δ*v* can be safely neglected.

Next, the experimental ratio *B*_p_/*B*_n_∼0.1 is about 10 times larger than *B*_Stark_/*B*_n_=0.009 estimated by Eq. (12). That is, the solvent Stark effect is expected to contribute about 10 % of the observed shift Δ*v*_p_ in polar solvents.

Consider now the shifts of the *S*_0_→*S*_n_ band (n=6 from our calculations) displayed in the middle frames of Figure 2. Here the nonpolar slope *B*_n_=(6560±190) cm^−1^ is twice steeper than that for the *S*_0_→*S*_1_ transition, in approximate agreement with the calculated higher polarizability in *S*_n_ (compared to *S*_1_, see Table 1). Turning to the polar slope *B*_p_=(190±40) cm^−1^, we note that it is 1.5 times *smaller* than that for the *S*_0_→*S*_1_ transition, contrary to what is expected if the slope would depend on the polarizability. Hence the polar and nonpolar shifts in anthracene are of different nature, consistent with the above assumption that Δ*v*_p_ originate mainly from the solute quadrupole (rather than from the solute polarizability).

**Figure 3:**
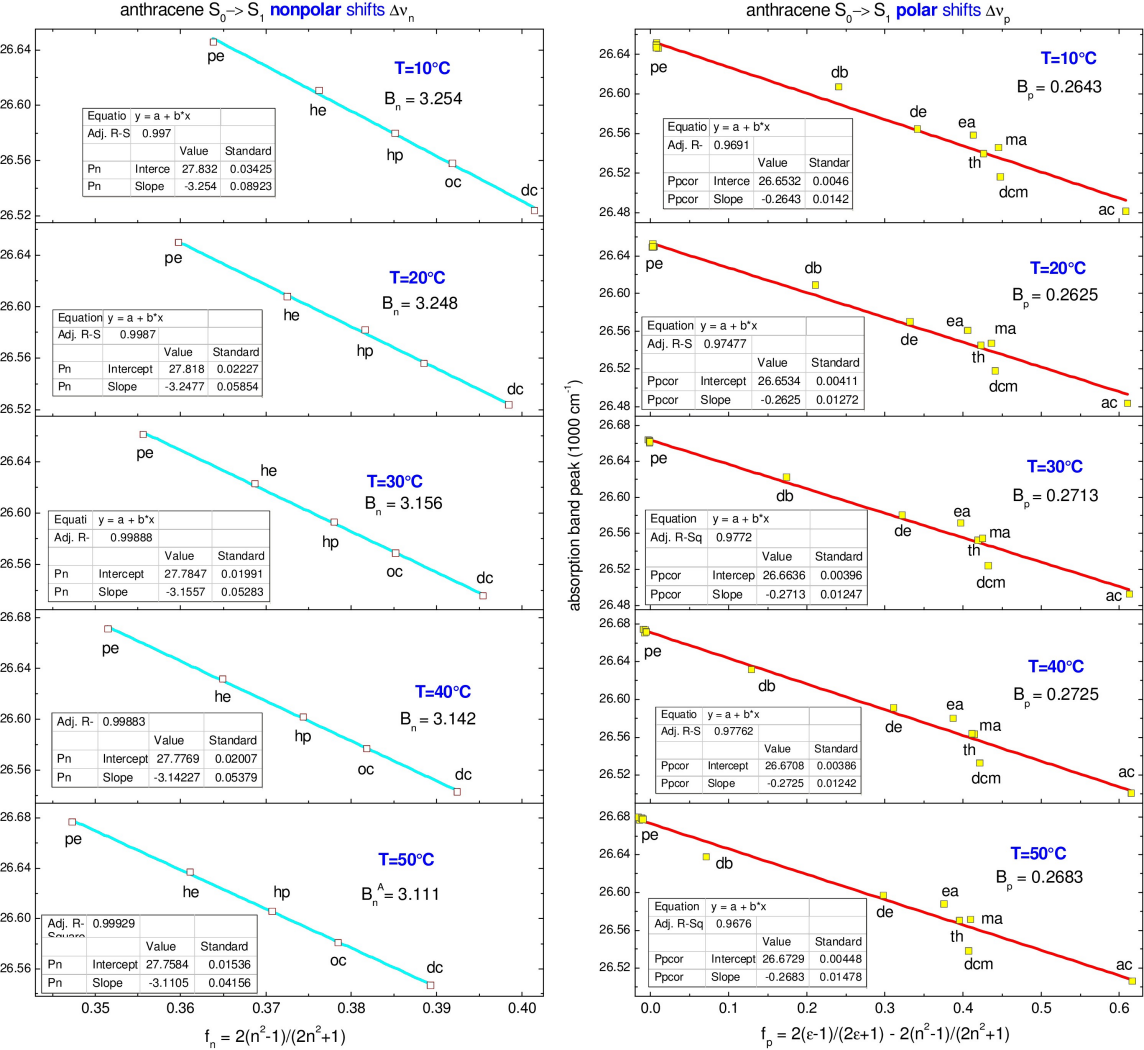
Temperature‐dependent nonpolar Δ*v*_n_(*T*) and polar Δ*v*_p_(*T*) shifts for the *S*_0_→*S*_1_ band of anthracene.

To isolate the Stark shift Δ*v*_Stark_ we measure the *S*_0_→*S*_1_ absorption spectra of anthracene at different temperatures. The results are presented in Figure 3 with nonpolar shifts Δ*v*_n_(*T*) shown at left and polar shifts Δ*v*_p_(*T*) at right, the corresponding slopes *B*_n_, *B*_p_ being indicated as inserts.

**Figure 4:**
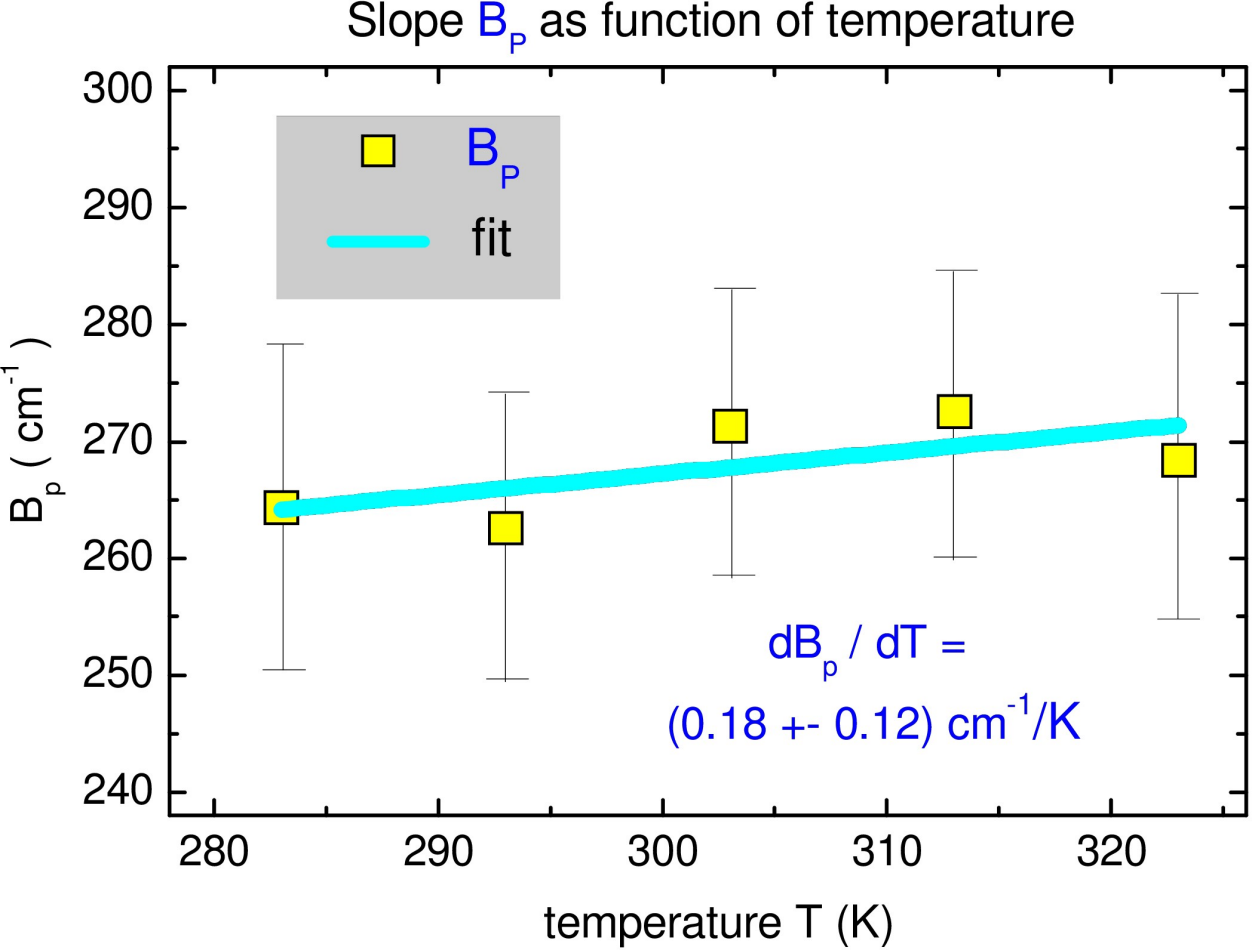
Temperature dependence of polar slope *B*_p_(*T*). Despite large error bars, the fit gives d*B*_p_/d*T*=(0.18 ±0.12) cm^−1^/K, in agreement with dielectric continuum theory, d*B*_Stark_/dT=0.096 cm^−1^/K (Eq. (12)). This corresponds to *B*_Stark_=(53±35) cm^−1^ at T=293 K. Directly measured shifts Δ*v*(T), without subtracting the nonpolar contribution, result in dB_p_/dT=(−0.07±0.14) cm^−1^/K (Figure S4), implying no apparent temperature dependence. Hence the subtraction of the nonpolar contribution is crucial when the shifts in polar solvents are modest in magnitude.

Figure 4 summarizes our results on the temperature‐dependent shifts. Here the slope *B*_p_(*T*) is shown as function of temperature. Despite large error bars, the fit gives d*B*_p_/d*T*=(0.18±0.12) cm^−1^/K, in good agreement with d*B*_Stark_/dT=0.096 cm^−1^/K (Eq. (12)) estimated by the dielectric continuum theory. We therefore ascribe the temperature dependence in Figure 4 to the Stark contribution that corresponds to *B*_Stark_=(53±35) cm^−1^ at T=293 K.

It is worth noting that *directly* measured shifts Δ*v*(T), without subtracting the nonpolar contribution, result in dB_p_/dT=(−0.07±0.14) cm^−1^/K (see Figure S4), that means in fact no actual temperature dependence. Thus, the subtraction of the nonpolar contribution is crucial when the polar shifts are modest in magnitude.

## 3 Conclusion

In summary, we derived the Stark shift of the *S*_0_→*S*_1_ band of anthracene from temperature‐dependent solvatochromic absorption shifts. The obtained derivative of the Stark slope d*B*_Stark_/dT=(0.18±0.12) cm^−1^/K corresponds to *B*_Stark_=(53±35) cm^−1^ at T=293 K, that constitutes approximately 10–20 % of the full slope *B*_p_ in polar solvents observed for anthracene and many other nondipolar chromophores. The measured Stark shift is in good agreement with the estimate from dielectric continuum theory.

To calculate the true shifts Δ*v*_p_ in polar solvents, it is necessary to subtract from directly measured shifts Δ*v* the nonpolar contribution which can be precisely determined by solvatochromic measurements in nonpolar solvents. The subtraction is especially necessary when the shifts Δ*v*_p_ are modest in magnitude that is usually the case for nondipolar or weakly polar chromophores.

## Experimental Section

Absorption spectra of anthracene in solution are recorded at *T*=10, 20, 30, 40, 50 °C, by spectrometer Cary 300 (Varian) with 0.2 nm step.

## Conflict of interest

The authors declare no conflict of interest.

## Supporting information

As a service to our authors and readers, this journal provides supporting information supplied by the authors. Such materials are peer reviewed and may be re‐organized for online delivery, but are not copy‐edited or typeset. Technical support issues arising from supporting information (other than missing files) should be addressed to the authors.

Supplementary
