# Supplementary material for: Evaluating the Solvent Stark Effect from Temperature‐Dependent Solvatochromic Shifts of Anthracene
Source: Chemphyschem. 2020 Feb 28;21(7):610–5. doi: 10.1002/cphc.202000010 (PMC7187296; doi:10.1002/cphc.202000010)
Supplement: Supplementary file 1 — Supplementary [file CPHC-21-610-s001.pdf]

CHEMPHYSCHEM

Supporting Information

Evaluating the Solvent Stark Effect from Temperature-Dependent Solvatochromic Shifts of Anthracene

Timais Janz, Manuel Güterbock, Fabian Müller, Martin Quick, Ilya N. Ioffe, Florian A. Bischoff, and Sergey A. Kovalenko*© 2020 The Authors. Published by Wiley-VCH Verlag GmbH & Co. KGaA.
This is an open access article under the terms of the Creative Commons Attribution License, which permits use, distribution and reproduction in any medium, provided the original work is properly cited.

Supporting Information

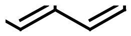

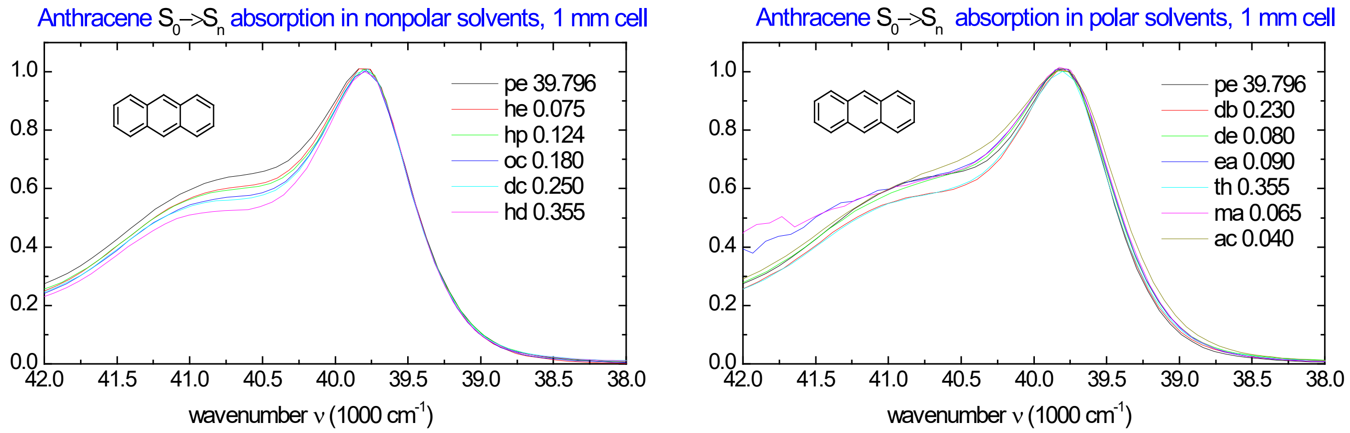

Fig. S1. Anthracene $S_0 \rightarrow S_n$ (n= 6 according to our calculations) absorption spectra at T=22°C. The spectra are shifted relative to n-pentane to achieve the best coincidence. The shifts are given as inserts.

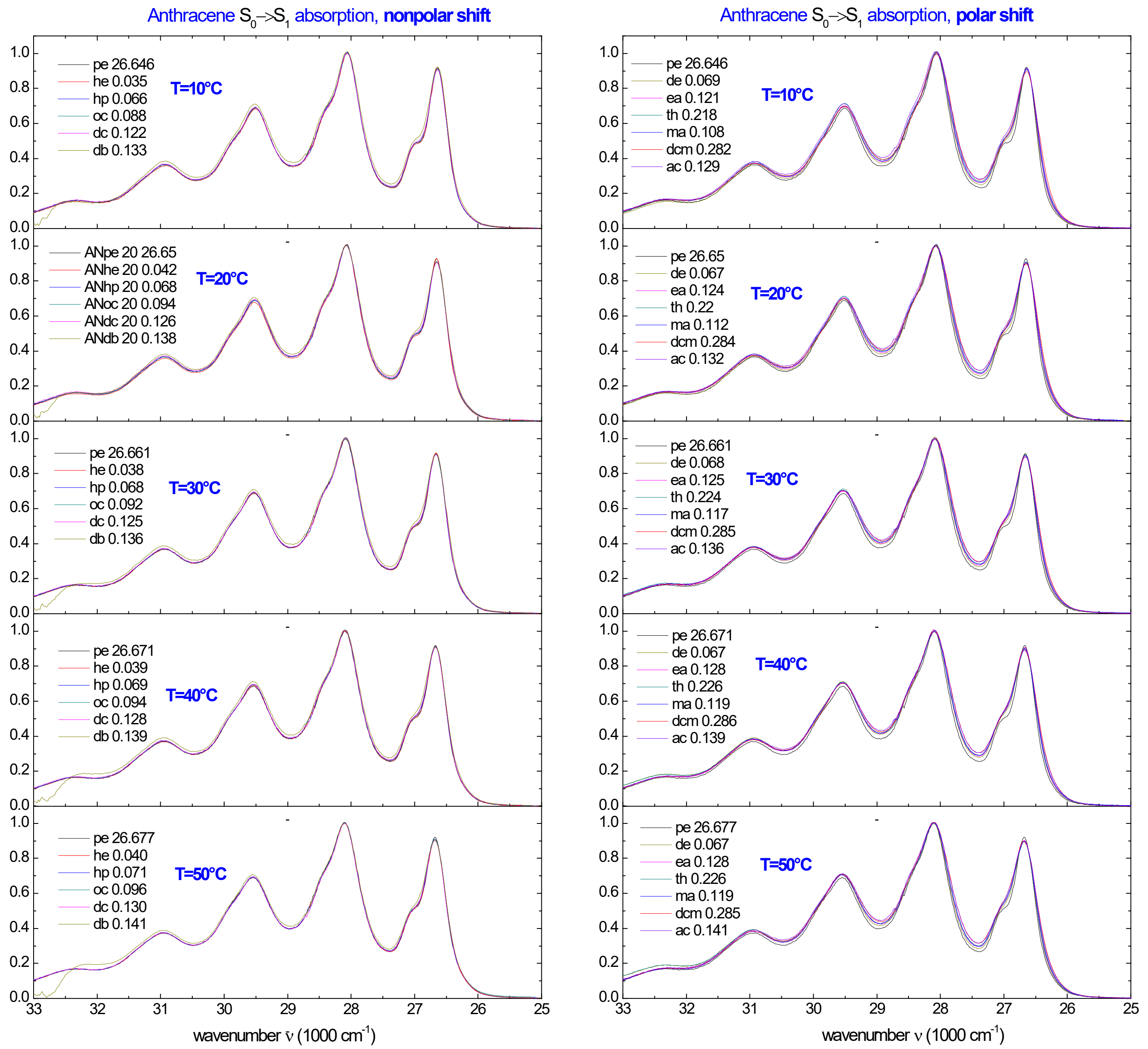

Fig. S2. Anthracene $S_0 \rightarrow S_1$ absorption spectra are shifted relative to n-pentane for best coincidence. The shifts indicated as inserts are determined with accuracy ±1 cm^{-1}.

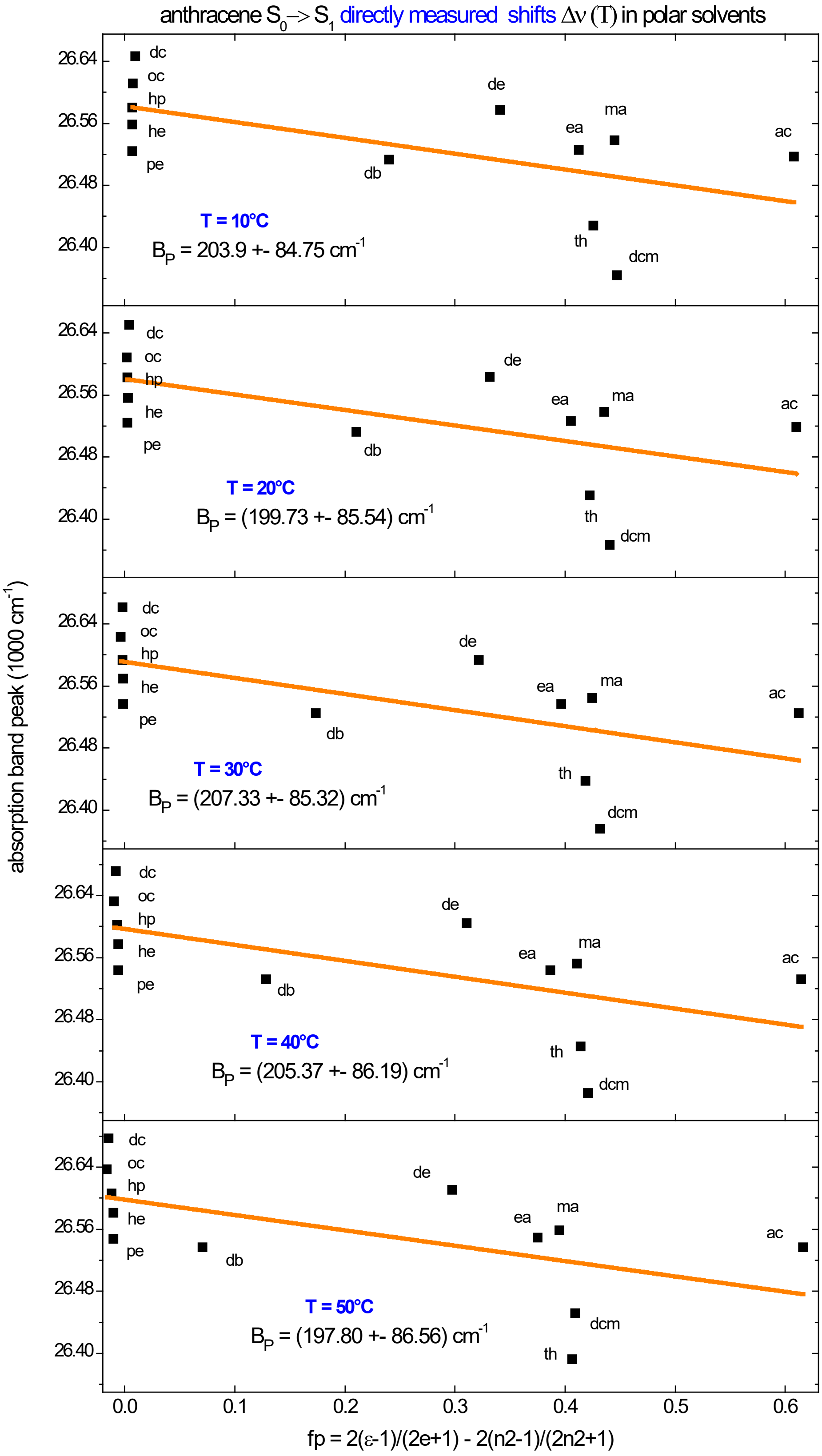

Fig. S3. Original temperature-dependent shifts $\Delta\nu(T)$ in polar solvents without subtracting the nonpolar contribution $\Delta\nu_n$ for the $S_0 \rightarrow S_1$ absorption band of anthracene. Linear fits result in the slopes $B_p(T)$ plotted in Fig. S4.

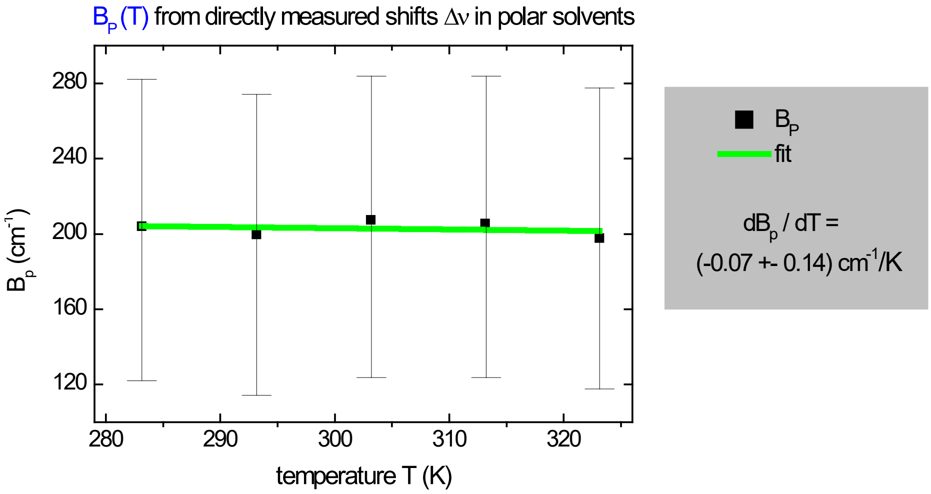

Fig. S4. Temperature-dependent slopes $B_p(T)$ from directly measured shifts $\Delta\nu(T)$ in polar solvents, shown in Fig. S3. The fit gives $dB_p/dT = (-0.07 \pm 0.14)$ cm^{-1}/K, that means no actual temperature dependence of the shifts. Hence the subtraction of the nonpolar contribution, $\Delta\nu_p = \Delta\nu - \Delta\nu_n$, is crucial for evaluating the solvent Stark effect on the solvatochromic shifts.
